# Continued 26S proteasome dysfunction in mouse brain cortical neurons impairs autophagy and the Keap1-Nrf2 oxidative defence pathway

**DOI:** 10.1038/cddis.2016.443

**Published:** 2017-01-05

**Authors:** Aslihan Ugun-Klusek, Michael H Tatham, Jamal Elkharaz, Dumitru Constantin-Teodosiu, Karen Lawler, Hala Mohamed, Simon M L Paine, Glen Anderson, R John Mayer, James Lowe, E Ellen Billett, Lynn Bedford

**Affiliations:** 1 School of Science and Technology, Nottingham Trent University, Nottingham, UK; 2 Centre for Gene Regulation and Expression, Sir James Black Centre, School of Life Sciences, University of Dundee, Dundee, UK; 3 Department of Pharmacology, Faculty of Medicine, University of Tripoli, Tripoli, Libya; 4 School of Life Sciences, University of Nottingham, Nottingham, UK; 5 Nottingham University Hospitals NHS Trust, Queen's Medical Centre, Nottingham, UK; 6 Department of Histopathology, Great Ormond Street Hospital for Children NHS Trust, London, UK; 7 School of Medicine, University of Nottingham, Nottingham, UK

## Abstract

The ubiquitin–proteasome system (UPS) and macroautophagy (autophagy) are central to normal proteostasis and interdependent in that autophagy is known to compensate for the UPS to alleviate ensuing proteotoxic stress that impairs cell function. UPS and autophagy dysfunctions are believed to have a major role in the pathomechanisms of neurodegenerative disease. Here we show that continued 26S proteasome dysfunction in mouse brain cortical neurons causes paranuclear accumulation of fragmented dysfunctional mitochondria, associated with earlier recruitment of Parkin and lysine 48-linked ubiquitination of mitochondrial outer membrane (MOM) proteins, including Mitofusin-2. Early events also include phosphorylation of p62/SQSTM1 (p62) and increased optineurin, as well as autophagosomal LC3B and removal of some mitochondria, supporting the induction of selective autophagy. Inhibition of the degradation of ubiquitinated MOM proteins with continued 26S proteasome dysfunction at later stages may impede efficient mitophagy. However, continued 26S proteasome dysfunction also decreases the levels of essential autophagy proteins ATG9 and LC3B, which is characterised by decreases in their gene expression, ultimately leading to impaired autophagy. Intriguingly, serine 351 phosphorylation of p62 did not enhance its binding to Keap1 or stabilise the nuclear factor erythroid 2-related factor 2 (Nrf2) transcription factor in this neuronal context. Nrf2 protein levels were markedly decreased despite transcriptional activation of the Nrf2 gene. Our study reveals novel insights into the interplay between the UPS and autophagy in neurons and is imperative to understanding neurodegenerative disease where long-term proteasome inhibition has been implicated.

Efficient protein degradation is crucial for proteostasis. The ubiquitin–proteasome system (UPS) and macroautophagy (autophagy) are the two major cellular catabolic pathways. The UPS is restricted to soluble proteins, whereas autophagy is involved in the degradation of a wider variety of substrates, including misfolded soluble proteins, protein aggregates and cellular organelles. Autophagy is known to compensate for impairment of the UPS and alleviate ensuing proteotoxic stress that impairs cell function.^1^ The UPS is regulated by a refined ubiquitin signalling system, tagging unwanted proteins with ubiquitin chains as a signal for their degradation by the 26S proteasome.^2^ Autophagic degradation also has the ability to be selective for its cargo via ubiquitin signalling, removing aggregated proteins, organelles and intracellular microbes.^3, 4, 5^ Selective autophagy is coordinated by autophagy receptors linking cargos tagged with ubiquitin chains to the autophagosomal membrane. p62/SQSTM1 (p62) is a well-characterised autophagy receptor, but additional receptors such as neighbour of BRCA1 gene 1 (NBR1), nuclear dot protein 52 kDa (NDP52) and Optineurin (OPTN) have been identified, and the regulatory mechanisms of selective autophagy are emerging.

Recent evidence demonstrates that phosphorylation of p62 at multiple sites specifies its function in selective autophagy. Phosphorylation of p62's ubiquitin-associated (UBA) domain at serine (S) 409 by unc-51-like autophagy-activating kinase 1 then S403 by TANK-binding kinase 1 and/or casein kinase 2 increases its binding affinity for ubiquitin, promoting translocation to the ubiquitinated cargo.^6, 7^ Following phosphorylation of the UBA domain of p62, S351 phosphorylation in the Kelch-like ECH-associated protein 1 (Keap1)-interacting region (KIR) by mammalian target of rapamycin complex 1 (mTORC1) takes place on ubiquitinated cargos. S351 phosphorylation of p62 increases its binding affinity for Keap1, competitively inhibiting Keap1's interaction with nuclear factor erythroid 2-related factor 2 (Nrf2) and sequestering Keap1 on autophagic cargos, coupling selective autophagy to activation of the Keap1–Nrf2 antioxidant pathway via stabilisation of Nrf2.^8, 9^ The transcription factor Nrf2 is normally constitutively degraded by the UPS; its binding partner Keap1 is a component of the E3 ubiquitin ligase complex that ubiquitinates Nrf2.^10^ Stabilised Nrf2 moves into the nucleus to activate the transcription of cytoprotective genes.^11^

Ubiquitin is found in the inclusion bodies in most neurodegenerative diseases, including Alzheimer's disease (AD), Parkinson's disease and amyotrophic lateral sclerosis.^12^ The characteristics of ubiquitin in this context remain unknown, but increasing evidence makes it plausible that UPS and autophagy dysfunctions cause aberrant accumulation of proteins and neurodegeneration.^13^ Aging is the major risk factor shared by sporadic neurodegenerative diseases and studies show cellular protein degradation systems become less efficient with age.^14, 15^ Recently, a role for the UPS in AD pathogenesis was firmly supported by pooled genome-wide association studies.^16^ Furthermore, conditional knockout studies in mice indicate that proteasomal and autophagic degradation have a causal link to neurodegenerative diseases in general.^17, 18, 19, 20^ Understanding the interplay between and contribution of the UPS and autophagy to proteostasis in neurons is imperative to understanding neurodegenerative disease. Here we investigate this in mouse brain cortical neurons with 26S proteasome dysfunction. We demonstrate short-term (early) 26S proteasome dysfunction induces selective autophagy, but long-term (continued) dysfunction decreases autophagy characterised by downregulation of essential autophagy genes. Furthermore, despite 26S proteasome dysfunction leading to S351 phosphorylation of p62 and early transcriptional activation of Nrf2, the Nrf2 protein is not stabilised in brain.

## Results

### 26S proteasome dysfunction in mouse forebrain neurons

26S proteasome dysfunction was restricted to mouse forebrain neurons by crossing floxed *Psmc1* mice (*Psmc1*^fl/fl^), an essential ATPase subunit of the 19S regulatory particle of the 26S proteasome, with mice expressing Cre recombinase under the control of the calcium calmodulin-dependent protein kinase II*α* (*CamKIIα*) promoter (*Psmc1*^fl/fl^;*CaMKIIα-Cre*) as described previously.^17^ Here ubiquitin immunostaining revealed sparse 26S proteasome-impaired neurons in *Psmc1*^fl/fl^;*CaMKIIα-Cre* cortices at 2 weeks old; significant accumulation of ubiquitinated proteins was evident from 3 weeks old (Supplementary Figure S1A), consistent with previous observations.^17^ The levels of synaptic and neuronal marker proteins were similar at 3 and 4 weeks old, but significantly decreased in *Psmc1*^fl/fl^;*CaMKIIα-Cre* cortices by 6 weeks old, reflecting progressive synaptic dysfunction and neurodegeneration (Supplementary Figure S1B).

### Mitochondrial fragmentation and mitophagy in 26S proteasome-impaired neurons

**Figure 1:**
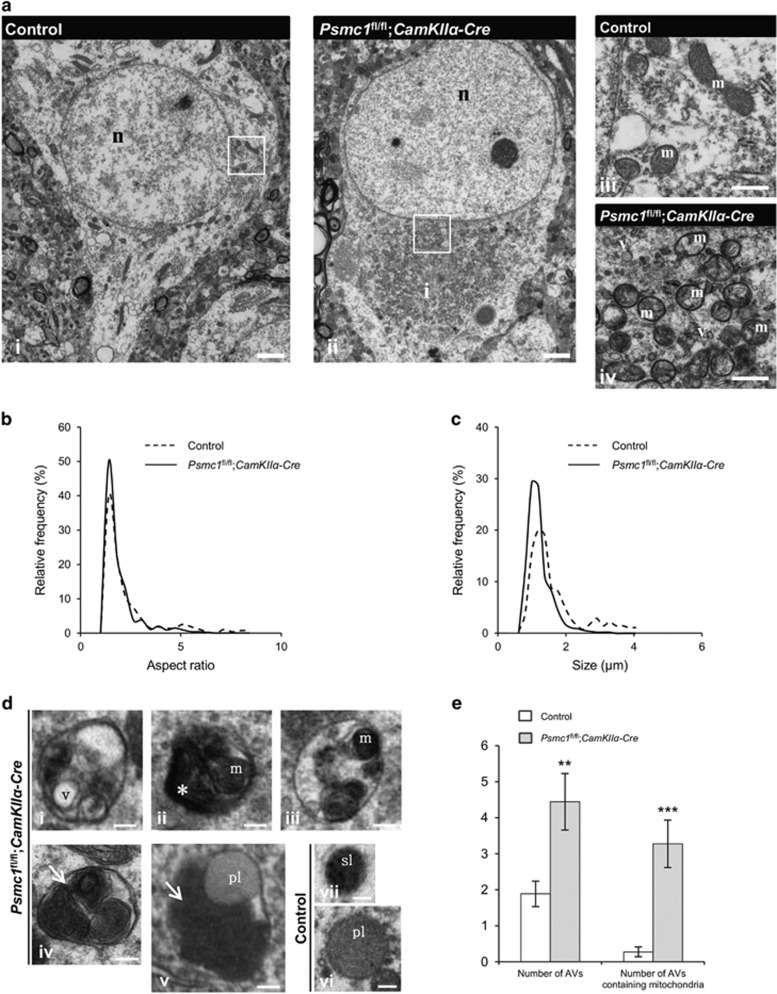
Mitochondrial fragmentation and mitophagy in 26S proteasome-impaired neurons. (**a**) Representative EMs of control (i) and *Psmc1*^fl/fl^;*CaMKIIα-Cre* (ii) cortical neurons at 6 weeks old. Enlarged views of mitochondria in boxed areas are shown in (iii, control) and (iv, *Psmc1*^fl/fl^;*CaMKIIα-Cre*). n, nucleus; i, inclusion; m, mitochondria; v, vesicle. Scale 2 *μ*m (i and ii) and 500 nm (iii and iv). (**b** and **c**) Smoothed line frequency histograms of mitochondrial aspect ratio and size distribution respectively for control and *Psmc1*^fl/fl^;*CaMKIIα-Cre* neurons. *P*<0.001 (**b**) and *P*<0.0001 (**c**) by Mann–Whitney test. (**d**) Representative AVs in 6-week-old *Psmc1*^fl/fl^;*CaMKIIα-Cre* cortical neurons (i–v) with heterogeneous intraluminal contents; vesicles (i; v), amorphous material (ii; asterisk) and condensed mitochondria (ii-v; m). Aggregates of condensed mitochondria resembling mitochondria undergoing autophagy are shown (iv and v; arrows). Representative primary (vi; pl) and secondary lysosomes (vii; sl) in 6-week-old control cortical neurons. Scale 200 nm. (**e**) Quantification of the total number of AVs and AVs containing mitochondria in 6-week-old control and *Psmc1*^fl/fl^;*CaMKIIα-Cre* neurons. Error bars represent S.E.M. **P<0.01 and ***P<0.001 by unpaired Student's *t*-test. Quantification in Figure 1 used 18 randomly selected neurons from three different control and *Psmc1*^fl/fl^;*CaMKIIα-Cre* brains

In a qualitative study, we previously described 26S proteasome dysfunction in mouse nigral neurons *in vivo* caused paranuclear accumulation of morphologically abnormal mitochondria.^21^ Here we show similar paranuclear accumulation of morphologically abnormal mitochondria in cortical neuron electron micrographs (EMs) and quantification analyses of mitochondria at 6 weeks old (Figure 1). Mitochondrial aspect ratio was significantly decreased in *Psmc1*^fl/fl^;*CaMKIIα-Cre* (median=1.41) compared to control (median=1.59) neurons, showing mitochondria were shorter (Figure 1b). Quantification of mitochondrial size showed mitochondria in *Psmc1*^fl/fl^;*CaMKIIα-Cre* (median=1.04 *μ*m) neurons were significantly smaller than in control (median=1.27* μ*m), confirming mitochondrial fragmentation (Figure 1c). Cytochrome oxidase IV (COXIV) qualitative immunostaining showed paranuclear accumulation of mitochondria from 3 weeks old, supporting early mitochondrial events following 26S proteasome dysfunction (Supplementary Figure S2A).

As the accumulation and fragmentation of mitochondria in 26S proteasome-impaired neurons is similar to that in models of mitophagy,^22, 23^ we investigated autophagy in cortical neuron EMs at 6 weeks old. Membrane-bound compartments resembling autophagic vacuoles (AVs) of diverse morphology (varied in size, content and electron density) corresponding to autophagosomes/autophagolysosome-like bodies were observed in 6-week-old *Psmc1*^fl/fl^;*CaMKIIα-Cre* neurons (Figure 1d i–v and Supplementary Figure S3). AVs were rarely observed in control neurons, but primary and secondary lysosomes were evident (Figure 1d vi and vii). Quantification revealed a significant increase in AVs and AVs containing condensed and aggregated mitochondria in *Psmc1*^fl/fl^;*CaMKIIα-Cre* compared to control neurons (Figure 1e). Furthermore, a temporal immunoblotting analysis of the mitochondrial inner membrane protein COXIV showed similar significantly decreased levels of COXIV at 4 and 6 weeks old in *Psmc1*^fl/fl^;*CaMKIIα-Cre* cortices, supporting early removal of mitochondria following 26S proteasome dysfunction (Supplementary Figure S2B). COXIV levels were also significantly decreased in *Psmc1*^fl/fl^;*CaMKIIα-Cre* cortices using urea lysis buffer, indicating that the mitochondria in the paranuclear aggregates were soluble in the total lysis buffer used (data not shown).

### Mitochondrial dysfunction in 26S proteasome-impaired neurons

**Figure 2:**
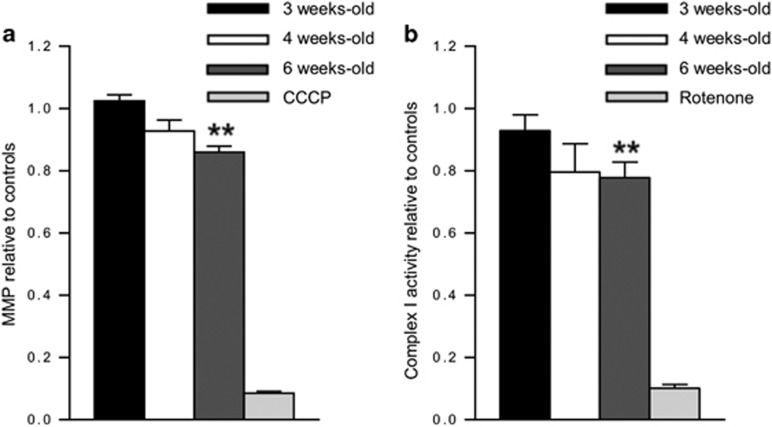
Mitochondrial dysfunction in 26S proteasome-impaired neurons. (**a**) Relative MMP of mitochondria purified from 3, 4 and 6-week-old control and *Psmc1*^fl/fl^;*CaMKIIα-Cre* cortices using JC1 potentiometric dye. MMP is calculated as the ratio red:green fluorescence. Protonophore carbonyl cyanide m-chlorophenyl hydrazine (CCCP) was used as a control; *n*≥3 mice. (**b**) Relative complex I activity of mitochondria purified from 3, 4 and 6-week-old control and *Psmc1*^fl/fl^;*CaMKIIα-Cre* cortices. Rotenone was used as a control; *n*≥8 mice. Error bars represent S.E.M. in (**a**) and (**b**). **P<0.01 by unpaired Student's *t*-test

To determine whether fragmented mitochondria in *Psmc1*^fl/fl^;*CaMKIIα-Cre* neurons were associated with functional impairments, we measured mitochondrial membrane potential (MMP) in freshly purified cortical mitochondria using JC1 dye. Mitochondrial depolarisation is evident by a decrease in red:green fluorescence ratio, which depends only on the membrane potential and not other factors such as mitochondrial size and shape. A significant decrease in MMP was evident by 6 weeks old in *Psmc1*^fl/fl^;*CaMKIIα-Cre* cortices compared with controls (Figure 2a). Further, we observed significantly decreased complex I activity in *Psmc1*^fl/fl^;*CaMKIIα-Cre* mitochondria at 6 weeks old, supporting mitochondrial dysfunction (Figure 2b).

### MOM protein degradation is inhibited by 26S proteasome dysfunction

**Figure 3:**
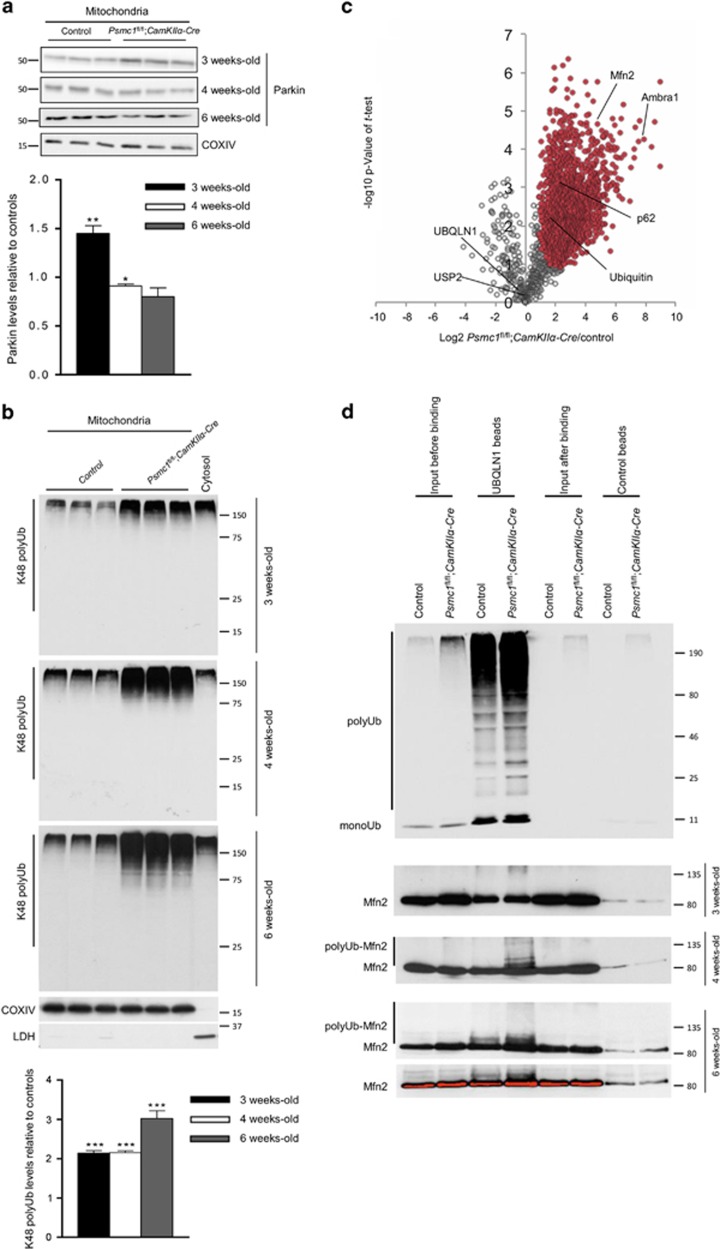
MOM protein degradation is inhibited by 26S proteasome dysfunction. (**a**) Western blots and quantification of Parkin on mitochondria purified from control and *Psmc1*^fl/fl^;*CaMKIIα-Cre* cortices at 3, 4 and 6 weeks old. COXIV was used as a loading control at each age for quantification; a representative COXIV is shown. Error bars represent S.E.M. of *n*=3 mice. *P<0.05 and **P<0.01 by unpaired Student's *t*-test. (**b**) K48 polyubiquitin (polyUb) Western blots of mitochondria purified from control and *Psmc1*^fl/fl^;*CaMKIIα-Cre* cortices at 3, 4 and 6 weeks old. Quantification used COXIV as a loading control at each age; a representative COXIV is shown. COXIV and LDH are shown for both mitochondria and cytosol to demonstrate mitochondrial purity. Threefold less cytosol was loaded to prevent overexposure with *α*-K48 ubiquitin. Efficiency of isolation procedure was also quantified by measuring mitochondrial and cytosol-specific marker enzymes citrate synthase and LDH respectively in total homogenate and then cytosol and mitochondria following the procedure (materials and methods). Error bars represent S.E.M. of *n*=3 mice. ***P<0.001 by unpaired Student's *t*-test. (**c**) Scatter plot showing log_2_ ratio of abundance of ubiquitinated proteins in 6-week-old control and *Psmc1*^fl/fl^;*CaMKIIα-Cre* cortices and -log10 *P*-value of *t*-test. In all, 1885 proteins were identified; 1504 were significantly different between experimental groups (red markers). Relative intensities of ubiquitin, Mfn2, p62 and Ambra1 are highlighted. UBQLN1 and USP2 shown for comparison. (**d**) UBQLN1 capture of ubiquitinated Mfn2 in control and *Psmc1*^fl/fl^;*CaMKIIα-Cre* cortices at 3, 4 and 6 weeks old using immunoblotting of independent samples from (**c**); *n*=3 mice. Specificity of the UBQLN1 pull-down is demonstrated using control beads without the UBQLN1 UBA domain. Although there is some Mfn2 binding to control beads this is not significant as shown by the pixel density (bottom image) at 6 weeks old and is similar for both control and *Psmc1*^fl/fl^;*CaMKIIα-Cre* cortices

Parkin-mediated lysine (K) 48-linked ubiquitination of mitochondrial outer membrane (MOM) proteins for 26S proteasomal degradation is important for efficient mitophagy.^22, 23^ Figure 3a shows Parkin on *Psmc1*^fl/fl^;*CaMKIIα-Cre* mitochondria is significantly increased at 3 weeks old, but decreased at 4 and 6 weeks old. Total levels of Parkin were not significantly different between control and *Psmc1*^fl/fl^;*CaMKIIα-Cre* cortices at any age (data not shown). Figure 3b shows significantly increased K48-linked ubiquitin chains associated with purified *Psmc1*^fl/fl^;*CaMKIIα-Cre* mitochondria from 3 weeks old, coinciding with Parkin recruitment and increasing 26S proteasome dysfunction. 26S proteasome complexes and activity gradually decrease between 2 and 4 weeks old in *Psmc1*^fl/fl^;*CaMKIIα-Cre* cortices.^17^ To further our investigations of ubiquitinated proteins on mitochondria we used immobilised UBA domain from Ubiquilin 1 (UBQLN1) to capture all ubiquitin chain linkage types from control and *Psmc1*^fl/fl^;*CaMKIIα-Cre* cortices at 6 weeks old followed by analysis via liquid chromatography tandem mass spectrometry (Supplementary Figure S4 and Supplementary Table S1). As expected, 26S proteasome dysfunction caused global accumulation of ubiquitinated proteins with most proteins showing increased abundance in *Psmc1*^fl/fl^;*CaMKIIα-Cre* mice (Figure 3c). In all, 217 proteins identified mapped to proteins in the mouse MitoCarta inventory, highlighting a wide mitochondrial ubiquitome (Supplementary Table S1). Specifically, we identified 46% of the class 1 candidate Parkin-dependent targets revealed by di-glycine profiling experiments and 21 known MOM ubiquitination targets associated with the depolarisation- and Parkin-dependent ubiquitome, including Mfn2 (Supplementary Table S1).^24, 25^ Figure 3d shows increased ubiquitinated Mfn2 in *Psmc1*^fl/fl^;*CaMKIIα-Cre* cortices from 4 weeks old. Furthermore, di-glycine capture proteomics showed K416 and K84 residues of Mfn2 were ubiquitinated as previously described in the seminal study by Sarraf *et al.*^25^ only in mitochondria purified from *Psmc1*^fl/fl^;*CaMKIIα-Cre* cortices, not control (Supplementary Table 1). Consistent with previous studies, we identified increased ubiquitinated autophagy receptors in *Psmc1*^fl/fl^;*CaMKIIα-Cre* cortices, including p62, NBR1 and Tax1-binding protein 1 (Supplementary Table S1, Supplementary Figures S4 and S5).^22, 24, 25^ Interestingly, ubiquitinated Ambra1 was one of the most significantly increased proteins in *Psmc1*^fl/fl^;*CaMKIIα-Cre* cortices captured by UBQLN1 (Supplementary Table S1,Supplementary Figures S4 and S5). Ambra1 has a role in the induction of autophagy as a component of the Beclin1 (BECN1)-phosphoinositide 3-kinase complex and proteasomal degradation of Ambra1 was recently shown to inhibit the induction of autophagy.^26, 27^

### 26S proteasome dysfunction induces selective autophagy, but continued dysfunction decreases essential autophagy proteins

**Figure 4:**
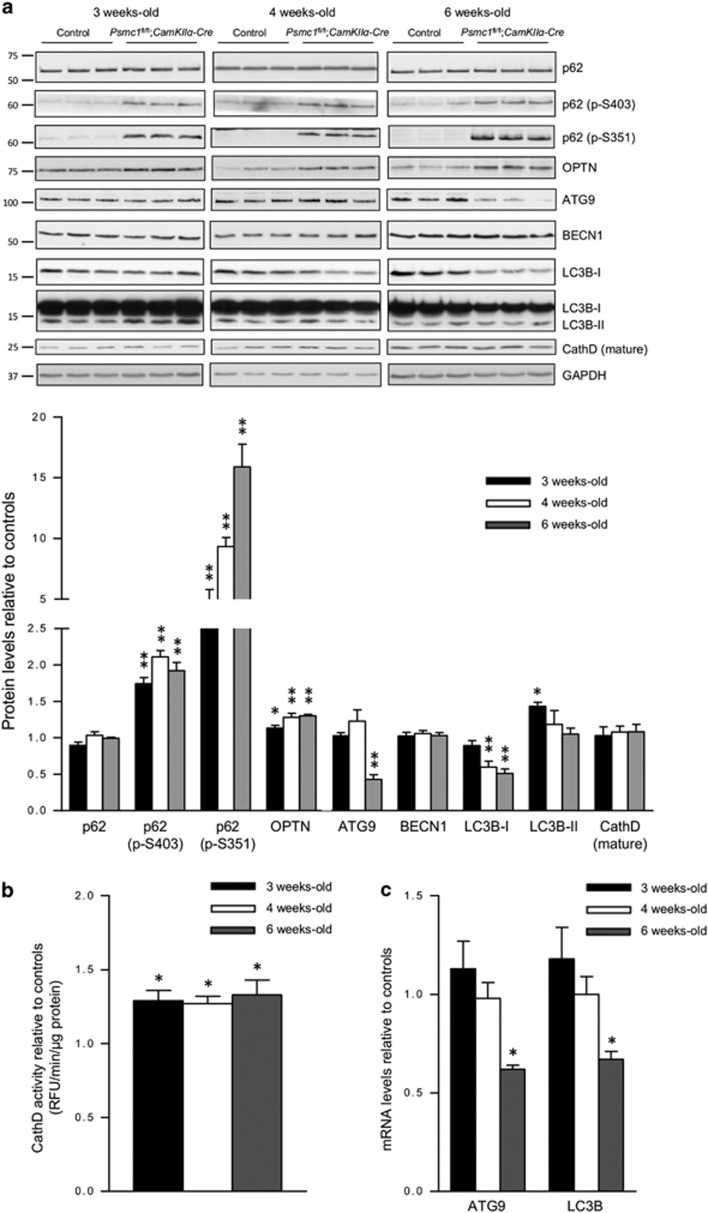
26S proteasome dysfunction induces selective autophagy, but continued dysfunction decreases essential autophagy proteins. (**a**) Representative immunoblots and quantification at 3, 4 and 6 weeks old of control and *Psmc1*^fl/fl^;*CaMKIIα-Cre* cortices. Short and long exposures for LC3B are shown. Glyceraldehyde 3-phosphate dehydrogenase (GAPDH) was used as a loading control at each age for quantification; a representative GAPDH is shown. Error bars represent S.E.M. of *n*≥3 mice. *P<0.05 and **P<0.01 by unpaired Student's *t*-test. (**b**) CathD activity in 3, 4 and 6-week-old control and *Psmc1*^fl/fl^;*CaMKIIα-Cre* cortices. Pepstatin A was used as a control and decreased CathD activity to zero values (data not shown). Error bars represent S.E.M. of *n*=5 mice. *P<0.05 by unpaired Student's *t*-test. (**c**) ATG9 and LC3B gene expression in control and *Psmc1*^fl/fl^;*CaMKIIα-Cre* cortices at 3, 4 and 6 weeks old. Error bars represent S.E.M. of *n*=8 mice. *P<0.05 by unpaired Student's *t*-test

As well as ubiquitin signalling, selective autophagy is regulated by molecular receptors such as p62 and OPTN.^3, 4, 5, 28^ Phosphorylation of p62 specifies its role as a selective autophagy receptor. Here we show that the total p62 protein levels were not significantly different between control and *Psmc1*^fl/fl^;*CaMKIIα-Cre* cortices, but the levels of S403 and S351 phosphorylated p62 were significantly increased in *Psmc1*^fl/fl^;*CaMKIIα-Cre* cortices from 3 weeks old, indicative of early induction of selective autophagy (Figure 4a). Figure 4a also shows significantly increased levels of OPTN in *Psmc1*^fl/fl^;*CaMKIIα-Cre* cortices from 3 weeks old, supporting selective autophagy induction. Levels of NBR1 were not significantly different between control and *Psmc1*^fl/fl^;*CaMKIIα-Cre* cortices (data not shown). We found elevated phosphorylated p62 and OPTN associated with mitochondria purified from *Psmc1*^fl/fl^;*CaMKIIα-Cre* cortices, substantiating the induction of mitophagy (Supplementary Figure S6). Increased levels of phosphorylated p62 and OPTN are also found in the cytosol and likely associated with protein aggregates that accumulate in 26S proteasome-impaired neurons as well (Supplementary Figure S6). S403 and S351 phosphorylated p62 displayed prominent perinuclear puncta in *Psmc1*^fl/fl^;*CaMKIIα-Cre* neurons at 3 weeks old (Supplementary Figure S7). At 6 weeks old, S403 phosphorylated p62 showed more diffuse staining associated with inclusions, whereas S351 phosphorylated p62 puncta were more prominent and localised to a single paranuclear area (Supplementary Figure S7).

To further investigate autophagy, we evaluated several essential proteins in control and *Psmc1*^fl/fl^;*CaMKIIα-Cre* cortices; ATG9, BECN1 and LC3B. Levels of ATG9 were markedly decreased at 6 weeks old in *Psmc1*^fl/fl^;*CaMKIIα-Cre* cortices (Figure 4a). BECN1 levels were not significantly different between control and *Psmc1*^fl/fl^;*CaMKIIα-Cre* cortices at any age (Figure 4a). LC3B-II was significantly increased at 3 weeks old in *Psmc1*^fl/fl^;*CaMKIIα-Cre* cortices, indicative of early induction of autophagy, but no significant differences were observed at 4 and 6 weeks old (Figure 4a). LC3B-I levels were similar at 3 weeks old, but significantly decreased in *Psmc1*^fl/fl^;*CaMKIIα-Cre* cortices at 4 and 6 weeks old, which together with decreased ATG9 suggests autophagy is decreased with continued 26S proteasome dysfunction (Figure 4a). Investigations of the lysosomal proteolytic enzyme cathepsin D (CathD) revealed that, despite similar levels of the mature protein (Figure 4a), CathD activity was significantly increased in *Psmc1*^fl/fl^;*CaMKIIα-Cre* cortices at 3, 4 and 6 weeks old (Figure 4b), suggesting this may be the result of a regulatory modification that enhances its proteolytic activity.^29^

LC3B-II is itself degraded by autophagy, which may explain some of the decrease in total LC3B protein levels in *Psmc1*^fl/fl^;*CaMKIIα-Cre* cortices, but ATG9 is thought to be recycled from the autophagosomal membrane.^30, 31^ To clarify whether the decreased levels of ATG9 and LC3B proteins were due to enhanced autophagic degradation or transcriptional regulation, we measured their gene expression in control and *Psmc1*^fl/fl^;*CaMKIIα-Cre* cortices. ATG9 and LC3B gene expression levels were significantly decreased in 6-week-old *Psmc1*^fl/fl^;*CaMKIIα-Cre* cortices, supporting downregulation of autophagy (Figure 4c).

### Continued 26S proteasome dysfunction in neurons impairs the Keap1-Nrf2 pathway

**Figure 5:**
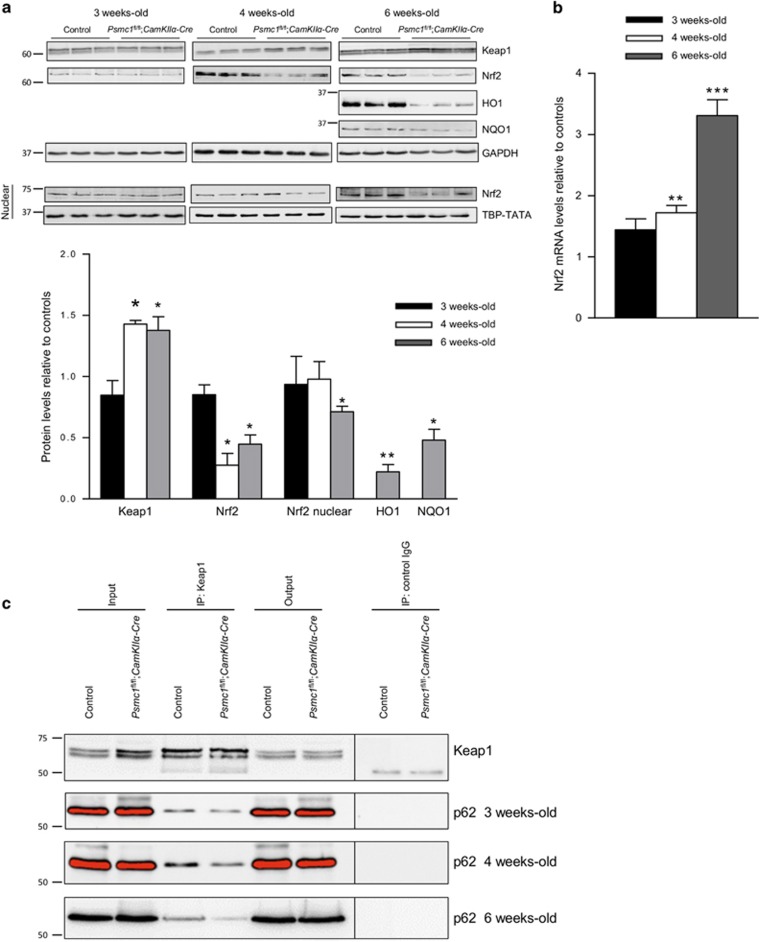
26S proteasome dysfunction in neurons impairs the Keap1-Nrf2 pathway. (**a**) Representative immunoblots and quantification at 3, 4 and 6 weeks old of control and *Psmc1*^fl/fl^;*CaMKIIα-Cre* cortices. Glyceraldehyde 3-phosphate dehydrogenase (GAPDH) was used as a loading control at each age for quantification; a representative GAPDH is shown. An immunoblot is also shown for Nrf2 in nuclear extracts at 3, 4 and 6 weeks old; TATA-binding protein TBP (TBP-TATA) was used as a loading control at each age for quantification. Error bars represent S.E.M. of *n*≥3 mice. *P<0.05 and **P<0.01 by unpaired Student's *t*-test. (**b**) Nrf2 gene expression in control and *Psmc1*^fl/fl^;*CaMKIIα-Cre* cortices at 3, 4 and 6 weeks old. Error bars represent S.E.M. of *n*=8 mice. **P<0.01 and ***P<0.001 by unpaired Student's *t*-test. (**c**) Immunoprecipitation of control and *Psmc1*^fl/fl^;*CaMKIIα-Cre* cortical homogenates at 3, 4 and 6 weeks old with Keap1 antibody followed by western blotting with Keap1 and p62 antibodies. The amount of Keap1 immunoprecipitated was similar in control and *Psmc1*^fl/fl^;*CaMKIIα-Cre* mice at 3, 4 and 6 weeks old; therefore, the Keap1 image (6-week-old) is representative of all ages

We recently showed 26S proteasome dysfunction in hepatocytes activated selective autophagy and the Keap1-Nrf2 antioxidant pathway via S351 phosphorylation of p62.^9^ Phosphorylated p62 and Keap1 proteins are degraded by autophagy. Here we found significantly increased levels of Keap1 protein at 4 and 6 weeks old in *Psmc1*^fl/fl^;*CaMKIIα-Cre* cortices by immunoblotting, suggesting impaired autophagic degradation (Figure 5a). This is supported by the marked increase in S351 phosphorylated p62 at 6 weeks old (Figure 4a). Comparable accumulation of phosphorylated p62 and Keap1 was not observed in mice with hepatocyte-specific knockout of *Psmc1*.^8, 9^ Unexpectedly, 26S proteasome dysfunction in neurons did not stabilise the Nrf2 protein. Nrf2 protein levels in *Psmc1*^fl/fl^;*CaMKIIα-Cre* cortices were significantly decreased in total extracts from 4 weeks old, as well as nuclear extracts at 6 weeks old (Figure 5a). Consequently, haem oxygenase-1 (HO-1) and NAD(P)H dehydrogenase quinone 1 (NQO1) proteins, major targets of Nrf2, were also markedly decreased at 6 weeks old (Figure 5a). However, we found Nrf2 gene expression was significantly increased in *Psmc1*^fl/fl^;*CaMKIIα-Cre* cortices at 4 and 6 weeks old (Figure 5b). To rationalise our observations, we investigated the binding of p62 and Keap1 in *Psmc1*^fl/fl^;*CaMKIIα-Cre* cortices by co-immunoprecipitation. Figure 5c shows less p62 co-immunoprecipitated with Keap1 in *Psmc1*^fl/fl^;*CaMKIIα-Cre* cortices, suggesting p62 phosphorylation does not enhance p62 binding to Keap1 in this *in vivo* neuronal context.

## Discussion

In this study, we show early induction of selective autophagy following 26S proteasome dysfunction *in vivo*, but with continued 26S proteasome dysfunction the levels of core autophagy proteins ATG9 and LC3 decreased associated with their decreased gene expression, which may be an adaptive response to prolonged autophagy induction. The increasing levels of autophagy substrates Keap1 and phosphorylated p62 with continued 26S proteasome dysfunction also support impaired autophagic degradation.^8, 32, 33^ Autophagy has a diverse range of protective roles, that is, during starvation, degradation of protein aggregates and dysfunctional organelles.^34, 35^ However, numerous studies have reported the notion of autophagic cell death (ACD) where over-activation of autophagy leads to excessive degradation of important cellular components and cell death.^36^ Therefore, the outcome may depend on the extent of autophagy induction and cellular context. Neurons may be sensitive to ACD because of their efficient autophagic flux.^37^ Autophagy is generally considered to compensate for proteasomal dysfunction and alleviate subsequent proteotoxic stress.^1^ These studies have been carried out in cellular models using pharmacological inhibitors of the 20S proteasome and involve short-term 20S proteasome inhibition. In our study, induction of autophagy following short-term (early) 26S proteasome dysfunction likely has a protective role, but continued 26S proteasome dysfunction may trigger excessive autophagy. The observed downregulation of essential autophagy genes provides a mechanistic response to terminate autophagy. Transcriptional regulation of ATG genes is a key aspect modulating autophagy activity.^38, 39^ Our results are supported by a recent study showing that sustained proteasome knockdown in primary culture and larval skeletal muscle inhibited autophagosome formation observed with short-term proteasome inhibition.^40^

Extensive work has focused on the PTEN induced putative kinase 1 (PINK1) kinase-Parkin ubiquitin ligase mitochondrial quality control pathway (mitophagy) that involves the UPS and autophagy.^41, 42^ Upon depolarisation, PINK1 is stabilised on the MOM where it recruits and activates cytosolic Parkin via an elaborate mechanism involving PINK1-dependent phosphorylation of ubiquitin and Parkin.^24, 43^ Parkin markedly changes the mitochondrial ubiquitome, promoting the assembly of ubiquitin chains of multiple linkage types on numerous mitochondrial proteins, some for 26S proteasomal degradation. Our results show that the upstream events of mitophagy are activated in *Psmc1*^fl/fl^;*CaMKIIα-Cre* neurons. We suggest Parkin is recruited from the cytosol to dysfunctional mitochondria before or at 3 weeks old and moves back to the cytosol at later stages following ubiquitination of MOM proteins, including the known Parkin-dependent target Mfn2. In agreement with previous reports using 20S proteasome inhibitors, increasing 26S proteasome dysfunction in *Psmc1*^fl/fl^;*CaMKIIα-Cre* neurons with age inhibits the degradation of ubiquitinated MOM proteins and downstream events of mitophagy, that is, efficient removal of dysfunctional mitochondria.^22, 23^ Proteasomes are long-lived cell constituents and appear to be in excess.^44^ Our previous studies showed 26S proteasome complexes and activity gradually decrease between 2 and 4 weeks old in *Psmc1*^fl/fl^;*CaMKIIα-Cre* cortices.^17^ Therefore, residual 26S proteasome function between 3 and 4 weeks old may degrade ubiquitinated MOM proteins, including Mfn2, and contribute to the removal of some dysfunctional mitochondria at early stages. Studies in cultured cells have been instrumental in discovering the role of the PINK1/Parkin pathway in mitochondrial quality control. To our knowledge, only two previous studies have investigated this pathway in neurons *in vivo* using mouse models of mitochondrial dysfunction.^45, 46^ Pickrell *et al.* recently provided strong evidence that the PINK1/Parkin pathway is active in brain. Our study provides further evidence of a role for this pathway in neurons *in vivo* and 26S proteasomal degradation of at least some MOM proteins may have a direct role in autophagic degradation of mitochondria. It is possible that decreased levels of essential autophagy proteins with continued 26S proteasome dysfunction in neurons contributes to inefficient removal of mitochondria. Immunohistochemical investigations of Parkin, as well as PINK1 and phospho-ubiquitin investigations were unsuccessful because of inadequate antibodies available for mice.

The roles of and spatiotemporal relationships between the receptors that regulate selective autophagy is still unclear. Here we show 26S proteasome dysfunction in cortical neurons is associated with increased phosphorylated p62 and OPTN on mitochondria, which taken together with increased LC3B-II and removal of some mitochondria, is indicative of selective autophagic degradation of ubiquitinated cargo at early stages. However, we cannot exclude the possibility that a late block in the normal high flux through the autophagy pathway in neurons may also lead to increased LC3B-II at this stage. p62 links ubiquitinated cargo with autophagosomes via its UBA domain and LC3-interacting region (LIR), respectively. As p62 itself is degraded in autolysosomes, the levels of p62 are used as an index of autophagic status. However, p62 protein changes are often cell type and context dependent, and this may be complicated by transcriptional regulation of p62.^47^ We did not observe changes in the total levels of p62 protein in *Psmc1*^fl/fl^;*CaMKIIα-Cre* cortices and it is possible that p62 is transcriptionally regulated in neurons in this context. However, we show increased phosphorylation of p62's UBA domain, which has recently been confirmed to specify its function in selective autophagy by increasing its binding affinity for ubiquitin and regulating selective autophagic degradation of ubiquitinated proteins and mitochondria.^7, 48^ OPTN is a more novel autophagy receptor and was recently shown to function in PARKIN-dependent mitophagy, binding to ubiquitinated mitochondria via its ubiquitin binding in ABIN (A20 binding and inhibitor of NF-κB) and NEMO (NF-*κ*B essential modulator) domain and inducing autophagosome assembly via its LIR.^49^ We found elevated OPTN associated with mitochondria in *Psmc1*^fl/fl^;*CaMKIIα-Cre* cortices, which may indicate the induction of mitophagy.

Unexpectedly, despite the marked increase in S351 phosphorylated p62, we did not find evidence of enhanced p62 binding to Keap1 or stabilisation of the Nrf2 protein. Interestingly, although groups have shown strong evidence that p62 phosphorylation is involved in selective autophagy, phosphorylation of both the UBA and KIR has not been reported together. Our data together with previous studies shows UBA phosphorylation in response to proteotoxic stress and supports a role in the regulation of selective autophagy in neurons *in vivo*.^6, 7, 50^ S351 phosphorylation of the KIR of p62 is thought to occur downstream of S403 phosphorylation and increases affinity between p62 and Keap1, coupling the Keap1-Nrf2 and selective autophagy pathways.^8^ Recent studies describing activation of these pathways, including following *Psmc1* inactivation, have been in liver.^8, 9^ S403 phosphorylated p62 has not been reported in autophagy-deficient liver to our knowledge. Autophagy is a tissue-specific, context-dependent process and our data may be explained by physiological differences between hepatocytes and neurons. It is also possible that phosphorylation of both the UBA and KIR of p62 affects binding between p62 and Keap1. Further, p62 may be involved in other complexes in *Psmc1*^fl/fl^;*CaMKIIα-Cre* neurons that affect its interaction with Keap1.^51^

The Nrf2 antioxidant pathway is activated at the transcriptional level in brain following 26S proteasome dysfunction, but the levels of Nrf2, as well as its target proteins decrease. Proteostasis relies on delicate interplay between protein synthesis and degradation. Therefore, continued 26S proteasome dysfunction may attenuate protein synthesis. Studies have shown that proteasome inhibition has an impact on eukaryotic translation initiation factor 2*α* and mTORC1; two important regulators of protein synthesis, leading to repression of translation.^52, 53, 54^ Nrf2 protein levels may be post-transcriptionally regulated by microRNAs.^55^ miR-144, miR-28, miR-200 and miR-34 have all been shown to fine tune the Nrf2 pathway.^56^ Importantly, it is also possible that neuroprotective antioxidant defences other that Nrf2 may be activated in brain, which is a mixed cell population, in response to proteotoxic stress. We previously showed that the astrocytic antioxidant enzyme peroxiredoxin 6 has an important role in response to early oxidative stress caused by neuronal 26S proteasome dysfunction. Therefore, activation of Nrf2 may not be necessary in this context.^57^

Ubiquitin is found in the inclusions in most neurodegenerative diseases and increasing evidence makes it plausible that UPS and autophagy dysfunctions have a causal link.^13^ Therefore, understanding the interplay between and contribution of the UPS and autophagy to proteostasis in neurons is imperative to understanding neurodegenerative disease. Our study reveals novel insights into proteostasis in neurons; 26S proteasomal degradation regulates autophagy. Similar to previous studies, we also show that short-term 26S proteasome dysfunction activates autophagy, but continued dysfunction in neurons leads to impaired autophagy and ultimately proteostasis collapse and cell death. Our findings are significant in the context of human disease where long-term proteasome dysfunction has been implicated and the enhancement of proteasome activity may be a promising strategy for the treatment of disease.

## Materials and methods

### Mice

Generation of conditional 26S proteasome-deleted mice has been described in detail previously.^17^ Appropriate littermate mice were used as controls (*Psmc1*^fl/fl^;*CaMKIIα-Wt* and *Psmc1*^fl/wt^;*CaMKIIα-Wt*). All procedures were carried out under personal and project licences granted by the UK Home Office in accordance with the Animals (Scientific Procedures) Act 1986 and with ethical approval from the University of Nottingham Ethical Review Committee.

### Electron microscopy

Mice were perfusion-fixed with 0.9% saline followed by glutaraldehyde fixative suitable for EM. A JEOL JEM-1400 120 kV Transmission Electron Microscope (JEOL UK Ltd., Welwyn Garden City) with a Deben AMT XR80 digital camera (Deben UK Ltd., Bury St Edmunds, UK) was used. Mitochondrial length or aspect ratio was quantified as the ratio between the major and minor axes of the ellipse equivalent to the mitochondrion; the ratio has a minimum value of 1 when it is a perfect circle and the value increases when mitochondria become elongated.^58^ Quantification of mitochondrial size was measured as their perimeter. Measurements were performed using Image J (http://imagej.nih.gov/ij/).

### Purification of mitochondria

Mitochondria were purified from freshly dissected cortices and all steps were on ice. Each cortex was homogenised in ice-cold buffered sucrose solution (2 mM HEPES pH 7.4, 210 mM mannitol, 70 mM sucrose, 0.1 mM EDTA, 1% (v/v) protease inhibitor cocktail (Sigma-Aldrich, Gillingham, UK, P8340)) using a Dounce tissue grinder (Fisher Scientific, Loughborough, UK). This was termed the total homogenate. Total homogenates were centrifuged at 500 *g* for 10 min followed by a further 10-min centrifugation at 1000 *g* of the re-suspended pellet in buffered sucrose solution. The resulting pellet was termed the nuclear-enriched fraction. Supernatants were combined and centrifuged at 7000* g* for 10 min. The resulting supernatant was termed the cytosolic fraction. The pellet was re-suspended in buffered sucrose solution and centrifuged at 7000 *g* for a further 10 min; this was repeated and the supernatants discarded. The pellet was re-suspended in buffered sucrose solution and layered onto a sucrose gradient of 60, 32, 23 and 15% sucrose in 10 mM MOPS (pH 7.2), 1 mM EDTA. Gradients were centrifuged at 130 000 *g* for 60 min at 4 °C and mitochondria were collected from the 32–60% interface and centrifuged at 10 000 *g* for 10 min at 4 °C to pellet the mitochondria. Pellets were then washed twice with buffered sucrose solution before re-suspending in buffered sucrose solution for storage; all fractions were stored at −80 °C.

The efficiency of mitochondrial purification was quantified by measuring the activity of mitochondrial and cytosol-specific marker enzymes citrate synthase and lactate dehydrogenase (LDH), respectively, in total homogenate and then cytosol and mitochondria following the procedure. This consistently showed approximately fivefold purification of mitochondria. We also performed COXIV and LDH western blot assays.

### Pull-down assay

Bacterial expression plasmids (pGEX-4T-1) encoding GST-UBA of UBQLN1 (VRFQQQLEQLSAMGFLNREANLQALIATGGDINAAIERLLGS) were kindly provided by Dr. Robert Layfield (Nottingham University, Nottingham, UK). Fusion proteins were expressed in XL-10 *Escherichia coli* strain, purified using glutathione sepharose beads (GE Healthcare, Amersham, UK, 17-0756-01) and UBQLN1 protein released by thrombin (Sigma-Aldrich T6884) cleavage. Purified UBQLN1 was then coupled to cyanogen bromide activated sepharose-4B beads (Sigma-Aldrich, C9142) in coupling buffer (100 mM NaHCO_3_, 500 mM NaCl, pH 8.3) followed by blocking with 1 M ethanolamine (pH 8). Control beads with no protein coupled were also prepared in parallel. For the pull-down assay, cortices were homogenised in buffer containing 50 mM Tris, 150 mM NaCl, 0.5% (v/v) Igepal, 5 mM *N*-ethylmaleimide, pH 7.5, 0.1% (v/v) protease and phosphatase inhibitors (Sigma-Aldrich, P5726) and 20 *μ*M MG-132 (Enzo Life Sciences, Exeter, UK, BML-PI102) using a bead beater (MP bio FastPrep®-24, MP Biomedicals, UK). Samples were centrifuged at 16 000 *g* for 20 min at 4 °C. Protein concentration was determined using Bradford protein assay. Dithiothreitol was added to each sample to a final concentration of 10 mM. Typically 5 mg of protein homogenate was added to 30 *μ*l of beads and incubated overnight at 4 °C with gentle mixing. Beads were then washed six times for 10 min each with 20 volumes of wash buffer (50 mM Tris, 150 mM NaCl and 0.5% (v/v) Igepal, pH 7.5). For mass spectrometry (MS) analysis, beads were re-suspended in deubiquitinating enzyme USP2 elution buffer (USP2; 50 mM Tris-HCl, 1 mM DTT, pH 7.4) and incubated overnight at 37 °C. Beads were collected by centrifugation and supernatant transferred to an Eppendorf for MS analysis. For western blotting, interacting proteins were eluted directly from the beads in sodium dodecyl sulphate-polyacrylamide gels (SDS-PAGE) sample buffer.

### Label-free mass spectrometry-based proteomic analysis of UBQLN1-interacting proteins

Triplicate samples from the three experimental groups, control beads+*Psmc1*^fl/fl^;*CaMKIIα-Cre* cortices, UBQLN1 beads+control cortices and UBQLN1 beads+*Psmc1*^fl/fl^;*CaMKIIα-Cre* cortices, were fractionated by SDS-PAGE (NuPage 10% polyacrylamide, Bis-Tris with MOPS buffer) and tryptic peptides extracted.^59^ MS analysis was performed by a benchtop orbitrap mass analyser (Q Exactive, Thermo Fisher Scientific, Waltham, MA, USA) equipped with an integrated nano-electrospray (Easy-Spray Thermo Fisher Scientific) coupled to a nano-uHPLC system (Easy N-LC-1000 Thermo Fisher Scientific). Peptides were fractionated on a 50 cm x 75 *μ*m ID, PepMap RSLC C18, 2 *μ*m reverse phase column run at 45 ^o^C, fractionating over 150 min (exact details of gradient available upon request). Data were acquired in the data-dependent mode to automatically switch between MS and MS/MS acquisition. Full scan spectra (*m/z* 300–1800) were acquired in the orbitrap with resolution *R*=70 000 at *m/z* 200 (after accumulation to a target value of 1 000 000 or 20 ms). The 10 most intense ions were fragmented by HCD (resolution 17 500 at *m/z* 200 and target value of 1 000 000 or 60 ms). MS raw data files were processed by MaxQuant (www.maxquant.org).^60^ Owing to the extreme stringency of the ubiquitin purification procedure that uses a ubiquitin-specific affinity reagent (UBQLN1), and a ubiquitin-specific elution reagent (USP2), the control bead samples contained very low protein amounts. Furthermore, because *Psmc1* inactivation causes a massive global shift in protein ubiquitination, the vast majority of proteins in the *Psmc1*^fl/fl^;*CaMKIIα-Cre* UBQLN1 purifications were more abundant than in the control equivalents. These two facts meant that MaxLFQ intensities could not be used because of the LFQ algorithm being dependent on the majority of proteins not changing in abundance across samples. Raw intensity values were normalised by median ratios within groups. Only proteins with three intensity values reported in at least one of the experimental groups were carried forward for analysis. In addition, decoy proteins, those likely to be external contaminants and proteins identified only by modified peptides were rejected. This left 1885 proteins. Any proteins identified in the UBQLN1 pull-downs without three intensity values in one group, but with three in the other (such as three from *Psmc1*^fl/fl^;*CaMKIIα-Cre* samples, but only one in control), had zero intensity values replaced using Perseus (using parameters width 0.3 downshift 0.8, separately for each sample). These intensity values were used to calculate two samples *t*-test *P*-values comparing UBQLN1 pull-downs from *Psmc1*^fl/fl^;*CaMKIIα-Cre* and control samples. The average of three ratios and –log10 *P*-value of the *t*-test were used to create the volcano plot shown in the Figure 3.

### PTMScan ubiquitin remnant motif (K-ɛ-GG) kit

Di-glycine-modified lysine residues were captured from purified mitochondria using the PTMScan Ubiquitin Remnant Motif (K-e-GG) kit according to the methods provided by Cell Signalling (New England Biolabs, Hitchin, UK).

### Preparation of total protein extracts

Mouse cortices were homogenised in total lysis buffer (50 mM Tris, 150 mM NaCl, 2 mM EDTA, 1 mM MgCl_2_, 100 mM NaF, 10% glycerol, 1% Triton X-100, 1% Na deoxycholate, 0.1% SDS, 125 mM sucrose and 1% (v/v) protease inhibitor cocktail) or urea lysis buffer (30 mM Tris, 8 M urea, 4% CHAPS and 1% (v/v) protease inhibitor cocktail) using a bead beater (MP bio FastPrep-24). Samples were centrifuged at 10 000 *g* for 10 min at 4 °C. Protein concentration was determined using Bradford protein assay.

### Gel electrophoresis and western blotting

Equal protein was subjected to electrophoresis on 8, 10 or 12% SDS-PAGE. Separated proteins were transferred onto nitrocellulose membrane using the Bio-Rad wet blotting system (Biorad Laboratories Ltd., Hemel Hempstead, UK). Equal protein loading was assessed by staining with 0.05% copper(II) phthalocyanine in 12 mM HCl. Blotted membranes were blocked for 1 h in 5% dried skimmed milk in Tris-buffered saline containing 0.1% (v/v) Tween-20 and then incubated overnight at 4 °C with primary antibodies diluted in blocking solution. Membranes were washed and incubated for 1 h at room temperature with appropriate horseradish peroxidise-conjugated secondary antibodies (Sigma-Aldrich, A6154 or A4416) in blocking solution. Antibody binding was revealed with enhanced chemiluminescence (ECL; Pierce, ThermoFisher Scientific, Waltham, MA, USA, 32106) or ECL prime (GE Healthcare, Amersham, UK, 2236) western blotting detection reagent. Digital images were captured using FujiFilm LAS-3000 mini and band intensity was quantified using Aida software (version 4.27.039, Elysia-raytest GmbH, Straubenhardt, Germany). Appropriate loading controls were used for quantification as indicated in the figure legends.

### Primary antibodies

SNAP25 (GTX113839), NSE (GTX101553), LDH (GTX101416), NBR1 (GTX114539) and HO-1 (GTX101147) from GeneTex, Inc.(Nottingham, UK); PSD95 (clone 6G6-1C9, MA1-045) from ThermoFisher Scientific (Waltham, MA, USA); GAPDH (G9545) and LC3B (L7543) from Sigma-Aldrich; p62 (ab56416), Nrf2 (ab31163), NQO1 (ab34173), CathD (ab6313) and TATA-binding protein TBP (ab51841) from Abcam (Cambridge, UK); Mfn2 (NBP1-96547), ATG9A (NB110-56893), Ambra1 (NBP1-07124) and Beclin1 (NB500-249) from Novus Biologicals (Abingdon, UK); Keap1 (10503-2-AP) from Proteintech (Manchester, UK); COXIV (4844) and Parkin (4211) from Cell Signalling; p62-S403 (D343-3) and p62-S351 (PM074) from Medical and Biological Laboratories Co., Ltd (Nagoya, Japan); K48-linkage-specific polyubiquitin antibody (05-1308) from Millipore; OPTN (H-220) (sc99214) from Santa Cruz (Heidelberg, Germany).

### Immunohistochemistry

Mice were perfusion fixed with 0.9% saline followed by 4% paraformaldehyde in phosphate-buffered saline (pH 7.4). Fixed brains were processed to paraffin using chloroform as the clearing agent. Immunostaining was performed as directed in Vector Laboratories M.O.M Immunodetection (PK2200) or Vectastain Elite Rabbit IgG ABC kits (PK6101) (Vector Laboratories Ltd., Peterborough, UK). In all, 0.01 M citrate buffer containing 0.05% Tween-20 (pH 6) was used for antigen retrieval. Primary antibody incubation was for 1 h at room temperature. Antibody binding was revealed using 3,3'-diaminobenzidine substrate and sections were counterstained with hematoxylin.

### Mitochondrial membrane potential

MMP was measured in freshly purified cortical mitochondria using JC1 dye (Invitrogen, T-3168). Mitochondria were incubated in 210 mM sucrose, 20 mM KCl, 3 mM glycylglycine, 1 mM KH_2_PO_4_ and 0.5 mM MgCl_2_ containing 30 mM glutamate, 20 mM malate and 7.5 *μ*g/ml JC1 at 37 °C for 15 min in the dark. No stained samples were incubated in buffer without JC1. In all, 1 *μ*M CCCP (Sigma-Aldrich, Gillingham, UK, C2759) was added before incubation at 37 °C as a control for mitochondrial depolarisation. Following incubation, samples were washed with buffer and centrifuged at 10 000 g for 5 min at room temperature. The resulting mitochondrial pellet was re-suspended in buffer and fluorescent intensity analysed using a PHERAstar plate reader (BMG LABTECH, Aylesbury, UK). JC1 displays green (Ex 490 nm, Em 530 nm) and red (Ex 490 nm, Em 590 nm) fluorescence. JC1 forms aggregates exhibiting red fluorescence when taken up by mitochondria with high membrane potential. MMP was normalised to total mitochondrial protein.

### NADH coenzyme Q reductase (NQR/complex I) assay

In all, 10 *μ*l of mitochondrial suspension was added to 290* μ*l XNA (5 mM KH_2_PO_4_, 5 mM MgCl_2_.6 H_2_0 and 5 mg albumin) and 25 *μ*l XNB (5 mM KH_2_PO_4_, 5 mM MgCl_2_.6 H_2_0, 5 mg human serum albumin and 7.1 mg saponin). This was incubated on ice for 5 min. In total, 72 *μ*l of this mixture was added to 75 *μ*l of Reagent 7 (2.33 ml master mix, 100* μ*l coenzyme Q1 (6 mM; Sigma-Aldrich C7956), 20 *μ*l of Antimycin A (0.6 mg/ml; Sigma-Aldrich A8674) and 50* μ*l of KCN (20 mM)) then a blank was measured for 2 min at 340 nm. In all, 3 *μ*l NADH (7.5 mM) was added and the absorbance measured for 2 min at 340 nm. In total, 2 *μ*l rotenone (10% Sigma-Aldrich R8875) was added to inhibit complex I (control) and absorbance was measured for a final 2 min at 340 nm. NQR activity was calculated by adjusting for inhibition of complex I by rotenone and normalised to total mitochondrial protein.

### CathD activity

CathD activity was measured using the fluorometric kit from Abcam (ab65302) following the manufacturers protocol. Activity was normalised to total protein. Pepstatin A (Merck, Darmstadt, Germany) was used as a control to inhibit CathD activity.

### Real-time RT-PCR

Total RNA was extracted from approximately 25 mg of cortical tissue using Tri Reagent (Sigma-Aldrich) and subsequently quantified spectrophotometrically at 260 nm with RNA purity being determined as the ratio of 260/280 nm readings. Thereafter, first-strand cDNA synthesis was carried out according to standard methodology. Transcripts were quantified by TaqMan PCR using an ABI prism 7900 sequence detector (Applied Biosystems Inc., Foster City, CA, USA) with 2 *μ*l of cDNA, 18 *μ*M of each primer, 5 *μ*M probe and Universal TaqMan 2x PCR Mastermix (Roche). Each sample was run in duplicate. Primers and MGB TaqMan probes (Thermo Fisher Scientific) were chosen where possible such that probes span over exon–exon boundaries to avoid genomic amplification (Supplementary Table S2). Hydroxymethylbilane synthase was used as internal control, and all genes of interest were labelled with the fluorescent reporter 5-carboxyfluorescein. Thermal cycling conditions: 10 min at 95 °C, followed by 40 cycles at 95 °C for 10 s then 60 °C for 30 s. The relative gene expression in the *Psmc1*^fl/fl^;*CaMKIIα-Cre* group compared with the control was calculated using the 2^−ΔΔCt^ method.

### Statistical analysis

Statistical analyses and *P*-values are specified in the figure legends; *n*=biological replicates.
