# Supplementary material for: Continued 26S proteasome dysfunction in mouse brain cortical neurons impairs autophagy and the Keap1-Nrf2 oxidative defence pathway
Source: Cell Death Dis. 2017 Jan 5;8(1):e2531–. doi: 10.1038/cddis.2016.443 (PMC5386360; doi:10.1038/cddis.2016.443)
Supplement: Supplementary Information [file cddis2016443x1.docx]

**SUPPLEMENTAL FIGURE LEGENDS**

**Figure S1. 26S proteasome dysfunction in mouse cortical neurons causes accumulation of ubiquitin and neurodegeneration between 2 and 6 weeks-old.** (A) Horizontal brain sections immunostained with ubiquitin. No significant differences were observed in control brains; therefore the control image is representative of all ages. Insets show the region of cortex examined and a higher magnification image of the neuropathological finding. Scale bar = 100 µm. (B) Immunoblots and quantification for presynaptic (synaptosomal-associated protein 25; SNAP-25), postsynaptic (postsynaptic density protein 95; PSD95) and neuronal (neuron-specific enolase; NSE) marker proteins demonstrating progressive synaptic dysfunction and neurodegeneration between 3 and 6 weeks-old. 0.63-, 0.39- and 0.7-fold decrease in SNAP25, PSD95 and NSE respectively at 6 weeks-old. Error bars represent SEM of n = 4 mice. *p < 0.05 and **p < 0.01 by unpaired Students *t*-test.

**Figure S2. (A) 26S proteasome dysfunction in mouse cortical neurons causes paranuclear accumulation of mitochondria from 3 weeks-old.** Cytochrome oxidase IV (COXIV) immunostaining of control and *Psmc1*^fl/fl^;*CaMKIIα-Cre* cortical horizontal sections between 2 and 6 weeks of age. No significant differences were observed in control brains; therefore, the control image is representative of all ages. Insets show the region of cortex examined and a higher magnification image of the neuropathological finding. Asterisks indicate paranuclear mitochondrial accumulation. Scale 100 µm. **(B) Induction of mitophagy in 26S proteasome-impaired neurons.** Immunoblots and quantification of COXIV at 3, 4 and 6 weeks-old in control and *Psmc1*^fl/fl^;*CaMKIIα-Cre* cortices. Error bars represent SEM of n = 4 mice. **p < 0.01 by unpaired Students *t*-test.

Figure S3. Representative EMs of control and *Psmc1*^fl/fl^;*CaMKIIα-Cre* cortical neurons at 6 weeks-old. ‘m’ indicates paranuclear accumulation of morphologically abnormal mitochondria in *Psmc1*^fl/fl^;*CaMKIIα-Cre* cortical neurons. AVs corresponding to autophagosomes/autophagolysosome-like bodies (or 2^o^ lysosomes) are highlighted by orange dots. AVs containing mitochondria are highlighted by blue dots. 1^o^ lysosomes in control neurons are highlighted by green dots. Scale 2 µm.

**Figure S4.** (A) Schematic of the experimental setup for the proteomic analysis of ubiquitinated proteins in mouse brain cortices and example of a single lane from the Coomassie-stained gel. (B) Column chart showing average intensity values and standard deviations for USP2 (the elution enzyme), UBQLN1 (the affinity reagent) and ubiquitin in the three experimental groups. (C) Scatter plot showing 1885 proteins with all three intensity values reported for either the UBQLN1 pull-down from control cortices, UBQLN1 pull-down from *Psmc1*^fl/fl^;*CaMKIIα-Cre* cortices or both. Note the general trend of almost all identifications towards increased abundance in *Psmc1*^fl/fl^;*CaMKIIα-Cre* cortices. MaxQuant LFQ values were not used in this analysis as it assumes most proteins do not change between conditions. Note ubiquitin and UQBLN1 were detected in very small quantities in the control pull-downs probably by carry-over between samples. (D) Scatter plot showing the relationship between log_2_ intensity ratio UBQLN1/control beads and total intensity for 175 proteins with three intensity values reported in the control bead compared to the UQ1 pull-down from *Psmc1*^fl/fl^;*CaMKIIα-Cre* cortices. Note the general trend towards greater abundance in the UBQLN1 purifications, and the lack of large numbers of purification contaminants that would usually reside in the center of the plot. This indicates a very clean purification strategy. (E) Relative intensities of ubiquitin, Mfn2, p62 and Ambra1 in 6 week-old control and *Psmc1*^fl/fl^;*CaMKIIα-Cre* cortices. (**F**) Ingenuity Pathways Analysis (www.ingenuity.com) “canonical pathway” enrichment comparing changers (x-axis) with non-changers (y-axis). (**G**) Ingenuity Pathways Analysis “diseases and functions” enrichment comparing changers (x-axis) with non-changers (y-axis). There was a particular enrichment for proteins involved in the ubiquitin system itself; suggesting 26S proteasome dysfunction has feedback consequences on enzymes of the ubiquitination pathway.

**Figure S5.** Validation of UBQLN1 capture of ubiquitinated p62 and Ambra1 proteins in control and *Psmc1*^fl/fl^;*CaMKIIα-Cre* cortices at 3, 4 and 6 weeks-old using immunoblotting of independent samples from Figure **3C**, n = 3 mice. Specificity of the UBQLN1 pull-down is demonstrated using control beads without the UBQLN1 UBA domain. * indicates a non-specific band.

**Figure S6.** **Phosphorylated p62 and OPTN are associated with purified mitochondria in 26S proteasome-impaired neurons.** p62 and OPTN Western blots of representative mitochondria, cytosolic and total fractions following the mitochondrial purification procedure from control and *Psmc1*^fl/fl^;*CaMKIIα-Cre* cortices. COXIV and LDH were used as loading controls and to demonstrate mitochondrial purity.

**Figure S7.** Immunohistochemical staining of 3 and 6 week-old mouse cortices with p-S403 and p-S351 p62 antibodies. Scale 20 µm. To visualize the subcellular localization of phosphorylated p62, we carried out immunohistochemistry of 3 and 6 week-old mouse brain sections. S403 and S351 phosphorylated p62 showed similar distribution in *Psmc1*^fl/fl^;*CaMKIIα-Cre* cortical neurons at 3 weeks-old; the majority of neurons contained prominent perinuclear puncta (i-iii and vii-ix), however, some neurons also showed diffuse staining (iii). Punctate S403 phosphorylated p62 staining was less prominent at 6 weeks-old, but diffuse staining was associated with neuronal inclusions (iv-vi). The puncta in S351 phosphorylated p62-stained neurons were more prominent at 6 weeks-old and frequently more localized to a single perinuclear area (x-xii). Phosphorylated p62 was not detected in control brain.

**SUPPLEMENTAL TABLES**

**Table S1.** Excel workbook containing the MaxQuant output data and downstream data processing for the ubiquitinated protein label-free proteomics experiment shown in Figures 3, S4 and S5. The first worksheet briefly describes the contents of the others.

**Table S2.** Real-time RT-PCR Assay IDs from ThermoFischer Scientific.
