# Supplementary figures and images for: Continued 26S proteasome dysfunction in mouse brain cortical neurons impairs autophagy and the Keap1-Nrf2 oxidative defence pathway
Source: Cell Death Dis. 2017 Jan 5;8(1):e2531–. doi: 10.1038/cddis.2016.443 (PMC5386360; doi:10.1038/cddis.2016.443)

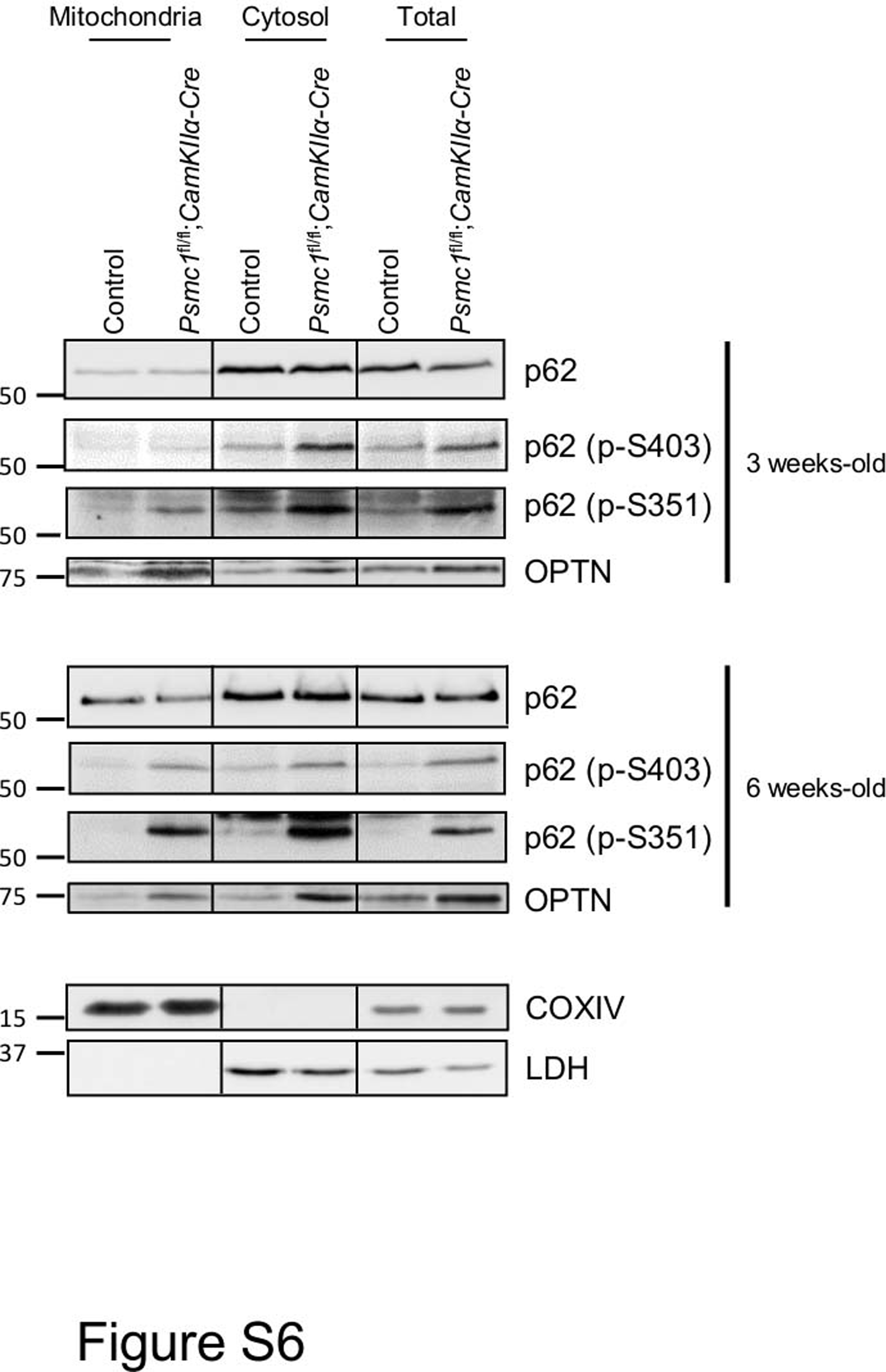

Supplement: Supplementary Figure S6 [file cddis2016443x8.tif]

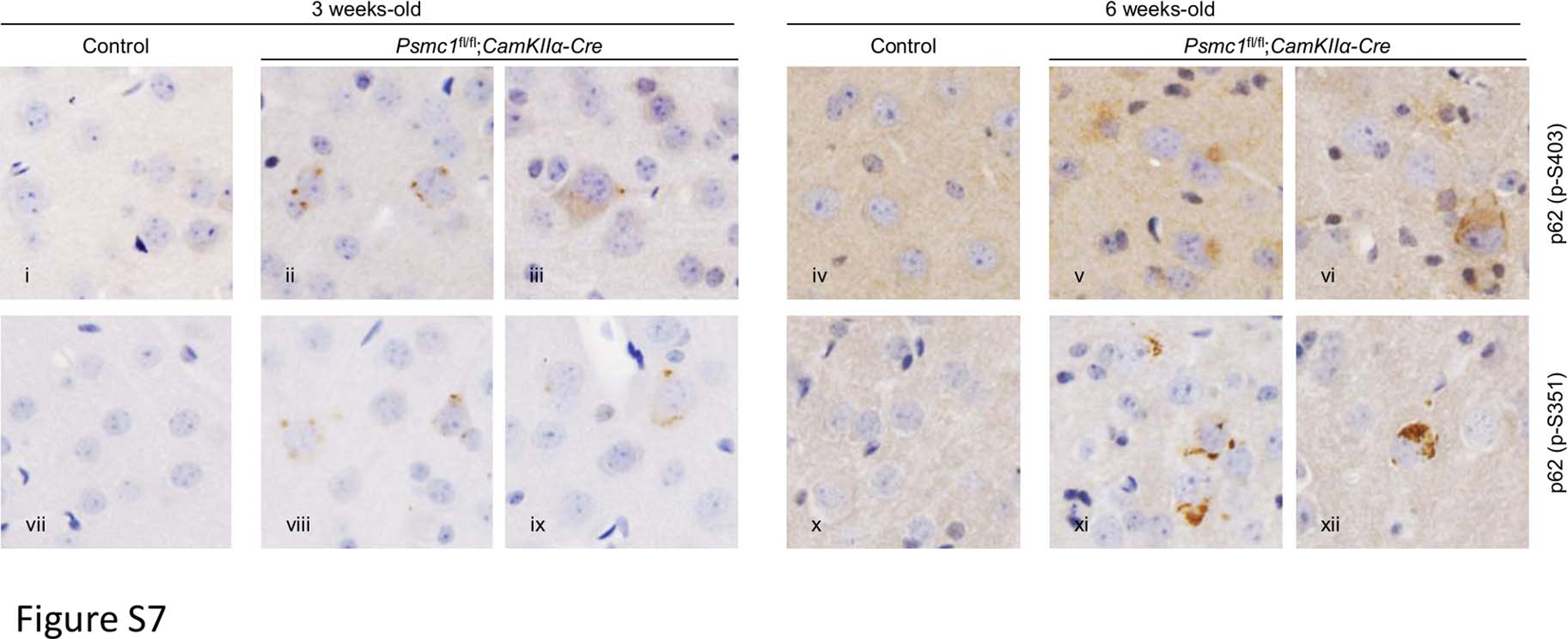

Supplement: Supplementary Figure S7 [file cddis2016443x9.tif]
