# Supplementary material for: Continued 26S proteasome dysfunction in mouse brain cortical neurons impairs autophagy and the Keap1-Nrf2 oxidative defence pathway
Source: Cell Death Dis. 2017 Jan 5;8(1):e2531–. doi: 10.1038/cddis.2016.443 (PMC5386360; doi:10.1038/cddis.2016.443)
Supplement: Supplementary Table S2 [file cddis2016443x11.docx]

**Supplementary Table S2** Real-time RT-PCR Assay IDs from ThermoFischer Scientific.

| **Gene Symbol** | **Gene name** | **Entrez Gene ID** | **ThermoFisher**  **Assay ID** | **Exon Boundary** | **Amplicon Size (bp)** |
| --- | --- | --- | --- | --- | --- |
| Atg9a | autophagy related 9A | 245860 | Mm01264420_m1 | 13 - 14 | 49 |
| Map1lc3b | microtubule-associated protein 1 light chain 3 beta | 67443 | Mm00782868_sH | 4 - 4 | 141 |
| Nfe2I2 | nuclear factor, erythroid derived 2, like 2 | 18024 | Mm00477784_m1 | 1 - 2 | 61 |
| Hmbs | hydroxymethylbilane synthase | 15288 | Mm01143545_m1 | 6 - 7 | 81 |
